# Synthetic biology applications in industrial microbiology

**DOI:** 10.3389/fmicb.2014.00451

**Published:** 2014-08-26

**Authors:** Weiwen Zhang, David R. Nielsen

**Affiliations:** ^1^Laboratory of Synthetic Microbiology, School of Chemical Engineering and Technology, Tianjin UniversityTianjin, China; ^2^Department of Chemical Engineering, School for Engineering of Matter, Transport, and Energy, Arizona State UniversityTempe, AZ, USA

**Keywords:** chemicals, microbial systems, synthetic biology, industrial microbiology, biotechnology

With the ability to perform a multitude of unique and complex chemical transformations, microorganisms have long been the “workhorses” of many industrial processes. However, in addition to exploiting the utility of naturally evolved phenotypes, the principles, strategies, and tools of synthetic biology are now being applied to facilitate the engineering of tailor-made microbes capable of tackling some of society's most important and toughest challenges. Fueled in part by exponentially increasing reservoirs of bioinformatic data and coupled with more robust and powerful tools for its processing, research in the past decade has brought about new and broadened perspectives of fundamental biological phenomena. The application of said insight is now beginning to unlock the unprecedented potential of synthetic biology in biotechnology, as well as its considerable promise for addressing previously unsolved global challenges. For example, within the realm of industrial microbiology, progress in the field of synthetic biology has enabled the development of new biosynthetic pathways for the production of renewable fuels and chemicals, programmable logic controls to regulate and optimize complex cellular functions, and robust microbes for the destruction of harmful environmental contaminants. In this Research Topic, a collection of articles—including original research, reviews, and mini-reviews—from leading investigators in the synthetic biology community are presented to capture the current state, recent progress, and over-arching challenges associated with integrating synthetic biology with industrial microbiology and biotechnology.

With improved tools and practices for engineering novel enzymes and pathways, synthetic biology has importantly expanded the diversity of chemical products that can now be synthesized biologically. These previously unattainable molecules serve important industrial roles as monomers for producing bioplastics (Adkins et al., 2012), bulk and fine chemicals, as well as long-chain alcohols (Lamsen and Atsumi, 2012) and other biofuels with improved “drop in” compatibility (Lee et al., 2013). Among those, as comprehensively reviewed by Prof. Tae Seok Moon and co-workers at Washington University in St. Louis, isoprenoids represent a diverse platform of molecules whose microbial production has been greatly enabled by improved understandings of complex regulatory and metabolic networks and the engineering of biological devices from well characterized and standardized genetic parts (Immethun et al., 2013). In fact, in an original study, the research group of Prof. Ganesh Sriram at the University of Maryland at College Park specifically demonstrate how the synthesis of one specific isoprenoid compound, namely dihydroartemisinic acid, by yeast can be improved by identifying non-trivial metabolic engineering targets through the tandem application of both *in silico* and *in vivo* metabolic pathway analyses (Misra et al., 2013).

Meanwhile, of equal importance to the product of interest is the microbial chassis used for its biosynthesis. In an original study, the research group of Prof. Ying-Jin Yuan of Tianjin University reports a strategy combining chassis selection and direct fine-tuning of the balance of key genes to increase ethanol yields in engineered xylose-utilizing *Saccharomyces cerevisiae* (Zha et al., 2012). In a continual push beyond the traditional platform microbes such as *Escherichia coli* and yeast, even cyanobacteria and algae^6^ are emerging as amenable platforms for rational engineering, as comprehensively reviewed by Prof. Deirdre Meldrum and co-workers at Arizona State University (Wang et al., 2012). By promoting the use of alternative sources of carbon and energy, including CO_2_ and sunlight in the latter case, these efforts are improving the sustainability of renewable product biosynthesis. In natural environments microorganisms commonly exist as communities of multiple species that are capable of performing more varied and complex tasks than in clonal populations. Prof. Travis Bayer and co-worker at Imperial College London review recent efforts to engineer synthetic microbial interactions of multiple microbial species (Brune and Bayer, 2012), emphasizing industrial applications in the field of biomining and bioremediation of acid mine drainage. In addition, Prof. Pier Luisi and co-workers at the University of Roma Tre present a comprehensive review on chemical synthetic biology, a new branch of synthetic biology oriented toward the synthesis of chemical structures alternative to those present in nature (Chiarabelli et al., 2013).

Two articles in this Research Topic focus more on the development and application of new enabling technologies in synthetic biology (Harrison and Dunlop, 2012; Reyes et al., 2012). The toxicity of biofuels to the producing microorganism is considered as one of the major challenges in achieving high-level fuel production. To address the issue, in an original research article, Prof. Mary Dunlop and co-worker at the University of Vermont develop a mathematical model for cell growth and biofuel production that implements a synthetic feedback loop using a biosensor to control efflux pump expression (Harrison and Dunlop, 2012). The results show that, in comparison to systems that express efflux pumps at a constant level, the feedback sensor increases overall biofuel production by delaying pump expression until it is needed. *In vitro* adaptive evolution has been used extensively for engineering various microbial systems, and the ability to identify the underlying adaptive landscape for a particular phenotype of interest will greatly enhance our abilities to engineer more robust microbial strains. Prof. Katy Kao and co-workers at Texas A&M University College Station present a comprehensive review on visualizing evolution in real-time (VERT), a recently developed methodology based on *in vitro* adaptive evolution that facilitates the identification of mutants with improved fitness throughout the course of evolution (Reyes et al., 2012).

Finally, Prof. Karmella Haynes and co-workers at Arizona State University discuss the importance of several issues surrounding risk mitigation and biocontainment. An important consideration as synthetic biology prepares to emerge from laboratory settings to enter full-scale biorefineries (Moe-Behrens et al., 2013).

As an emerging scientific field, synthetic biology is one of the most dynamic new fields of biotechnology in the past years. Its application in industrial microbiology, although still at very beginning, has demonstrated that it is a powerful technology that could significantly improve the industrial microbiology research to meet the challenges human society faces today.

## Conflict of interest statement

The authors declare that the research was conducted in the absence of any commercial or financial relationships that could be construed as a potential conflict of interest.

## References

[B1] Adkins J. Pugh S. McKenna R. Nielsen D. R. (2012). Engineering microbial chemical factories to produce renewable biomonomers. Front. Microbiol. 3:313 10.3389/fmicb.2012.00313 22969753 PMC3430982

[B2] Brune K. D. Bayer T. S. (2012). Engineering microbial consortia to enhance biomining and bioremediation. Front. Microbiol. 3:203 10.3389/fmicb.2012.00203 22679443 PMC3367458

[B3] Chiarabelli C. Stano P. Luisi P. L. (2013). Chemical synthetic biology: a mini-review. Front. Microbiol. 4:285 10.3389/fmicb.2013.00285 24065964 PMC3779815

[B4] Harrison M. E. Dunlop M. J. (2012). Synthetic feedback loop model for increasing microbial biofuel production using a biosensor. Front. Microbiol. 3:360 10.3389/fmicb.2012.00360 23112794 PMC3481154

[B5] Immethun C. M. Hoynes-O'Connor A. G. Balassy A. Moon T. S. (2013). Microbial production of isoprenoids enabled by synthetic biology. Front. Microbiol. 4:75 10.3389/fmicb.2013.00075 23577007 PMC3616241

[B6] Lamsen E. N. Atsumi S. (2012). Recent progress in synthetic biology for microbial production of C3-C10 alcohols. Front. Microbiol. 3:196 10.3389/fmicb.2012.00196 22701113 PMC3370425

[B7] Lee S. J. Lee S. J. Lee D. W. (2013). Design and development of synthetic microbial platform cells for bioenergy. Front. Microbiol. 4:92 10.3389/fmicb.2013.00092 23626588 PMC3630320

[B8] Misra A. Conway M. F. Johnnie J. Qureshi T. M. Lige B. Derrick A. M. (2013). Metabolic analyses elucidate non-trivial gene targets for amplifying dihydroartemisinic acid production in yeast. Front. Microbiol. 4:200 10.3389/fmicb.2013.00200 23898325 PMC3724057

[B9] Moe-Behrens G. H. Davis R. Haynes K. A. (2013). Preparing synthetic biology for the world. Front. Microbiol. 4:5 10.3389/fmicb.2013.00005 23355834 PMC3554958

[B10] Reyes L. H. Winkler J. Kao K. C. (2012). Visualizing evolution in real-time method for strain engineering. Front. Microbiol. 3:198 10.3389/fmicb.2012.00198 22661973 PMC3362087

[B11] Wang B. Wang J. Zhang W. Meldrum D. R. (2012). Application of synthetic biology in cyanobacteria and algae. Front. Microbiol. 3:344 10.3389/fmicb.2012.00344 23049529 PMC3446811

[B12] Zha J. Hu M. L. Shen M. H. Li B. Z. Wang J. Y. Yuan Y. J. (2012). Balance of XYL1 and XYL2 expression in different yeast chassis for improved xylose fermentation. Front. Microbiol. 3:355 10.3389/fmicb.2012.00355 23060871 PMC3464680

